# Impacts of noise pollution from high-speed rail and road on bird diversity: a case study in a protected area of Italy

**DOI:** 10.1007/s11356-024-33372-0

**Published:** 2024-04-20

**Authors:** Ester Bergamini, Sofia Prandelli, Fausto Minelli, Roberto Cazzolla Gatti

**Affiliations:** 1 Department of Biology, University of Pisa, Pisa, Italy; 2 Department of Biological, Geological, BIOME-Biodiversity and Macroecology Lab, and Environmental Sciences (BiGeA), University of Bologna, Bologna, Italy; 3 Ente di Gestione per i Parchi e la Biodiversità-Emilia Centrale, Modena, Italy; 4 Biological, Geological and Environmental Sciences Department (BiGeA), BIOME Lab, University of Bologna, Bologna, Italy

**Keywords:** Disturbance, Sound, Birds, Infrastructure, Railway, Highway, Biodiversity

## Abstract

The disturbance of infrastructures may affect biological communities that are exposed to them. This study assesses the impact of high-speed (highway and railway) infrastructures in a protected study site, the Natural Reserve Fontanili di Corte Valle Re (Emilia–Romagna, Italy). We compared bird diversity with sound intensity and frequency in three sampling areas, increasingly distant from the infrastructures at the border with the reserve, during the last 4 years (2019–2022), monitoring sedentary, nesting, and migratory bird species. We hypothesize a decreasing diversity closer to the source of disturbance, which is mostly attributable to noise pollution. Our findings confirmed this trend, and we show that, in particular, disturbance seems to influence species richness more than the total abundance of birds. We also discovered that highway disturbance was much higher than railway in terms of frequency and duration. In light of these results, we suggest that some species, which have a behavioral ecology strongly based on singing to communicate with each other for their reproductive and defensive strategies, may suffer more from constant acoustic disturbance. The installation of effective noise barriers to shield the sound produced by the highways should be considered a mandatory request not only in proximity to houses but also in the vicinity of protected areas.

## Introduction

Anthropogenic disturbance has been widely studied in ecology since its effects have consequences on communities and their composition (Ausprey et al. 2023). According to Pickett et al. (1989), disturbance can be defined as a change in the ecosystem caused by an external factor to the level of interest. Another well-accepted idea is that disturbance, as every process that causes loss of biomass in a community (Grime 1977), also has a negative impact on a community and its elements.

Previous studies showed that the proximity to disturbing infrastructures is one of the biggest causes of biodiversity loss (Van Der Zande et al. 1980). In particular, the number of negative effects of roads and railways is high when considering mortality due to vehicle collisions, particularly for reptiles, amphibians, and mammals. Although birds showed contrasting effects on infrastructure’s presence, the impact of the acoustic disturbance may be relevant for these taxa, even if poorly investigated. A recent study from Ausprey et al. shows how any kind of disturbance, in agricultural landscapes, leads to a decline in species and that sensitivities to anthropogenic disturbance are species-specific (Ausprey et al. 2023). The decrease in abundance and species diversity in birds is particularly documented at short distances, up to 1 km from the source of disturbance (Benítez-López et al. 2010). The disturbance from viable infrastructures is mostly acoustic and its consequences are proportional to the magnitude of the noise. There is preliminary evidence that birds are less affected by train passage, which is discontinuous than by the motor vehicles on the highways, which is quite constant (Wiącek et al. 2015). Species that possess communicative skills and base their reproductive behaviors mostly on vocalization are certainly the most affected (Lucas et al. 2017). A study conducted by Reijnen and Foppen (2006) showed that an important level of disturbance, such as big and busy roads, is the main cause of negative abundance trends of birds, compared to the presence of the infrastructure itself.

At the same time, there might be a positive effect that increases bird diversity due to the landscape’s heterogeneity created by roads and railways (Morelli et al. 2014; Kajazer-Bonk et al. 2019). Close to the infrastructure, woody habitats would be side by side with crops, leading to an increase in the amount of food availability. Moreover, the traffic could lead to a reduction in the number of predators, while artificial light would extend diurnal activities in winter. Having different habitats also means having more nesting sites and chances of hiding (Kajzer-Bonk et al. 2019).

This study aims to analyze the impact of acoustic disturbance produced by high-speed road and rail on bird diversity of migratory, nesting, and sedentary species of a protected area in northern Italy. We hypothesize that both species abundance and richness increase with incremental distance from the source of disturbance. On the other hand, we evaluated how the mid-domain effect could lead to a higher abundance and diversity in the core of the reserve and a decrease of them at the edge (Colwell and Lees 2000).

## Materials and methods

### Study site

The study was conducted in the bird-ringing site of Natural Reserve Fontanili di Corte Valle Re, in Italy. The site (coordinates 44.7672 N, 10.5328 E) has an area of 37 hectares and is located in the municipality of Campegine (Reggio Emilia). Data were collected at an increasing distance from two big infrastructures: the A1 highway and the high-speed railway Milano–Bologna, both adjacent to the protected area. The presence of specimens was studied for the reproductive and migratory seasons, from autumn 2019 to autumn 2022. The monitoring was done for the MonITRing Project of the National Centre of Ringing (CNI), of the Superior Institute for Environmental Protection and Research (ISPRA).

The protected area is one of the last flatland springs remaining in its natural state. Around the regional Reserve, water crosses cultivated fields and, near the springs, it leads to the growth of hydrophilic plants and reeds. The woods in which nets were located is mainly composed of black alder (*Alnus glutinosa*), grey sallow (*Salix cinerea*), alder buckthorn (*Rhamnus frangula*), black elderberry (*Sambucus nigra*), field elm (*Ulmus minor*), blackthorn (*Prunus spinosa*), English hawthorn (*Crataegus laevigata*), and dogwood (*Cornus sanguinea*). Different microhabitats allow the presence of a rich and varied fauna (Parchi Emilia Centrale 2022). The vulnerability of the Reserve, besides the rarity of its flatlands, is due to the closeness to the A1 highway, one of the busiest in Italy, and the adjacent high-speed railroad. The area is also part of the EU Natura 2000 network (Mézard et al., 2009) within the Area of ecological restoration Fontanili Media Pianura Reggiana and it is a Site of Community Importance (SCI), in which habitats are protected (Ambiente, Regione Emilia Romagna 2022).

### Sampling methods and data analysis

The catch of the specimens took place using mist nets that have a mesh size of 16 mm, height of 2.5 m, and width of 12 m. Four of them were placed in three spots hereafter denominated Near, Intermediate, and Far, for a total net length of 48 m in each of them. The three transects were all placed perpendicular to the viable infrastructures and in areas where plant cover and composition are mostly the same.

**Fig. 1:**
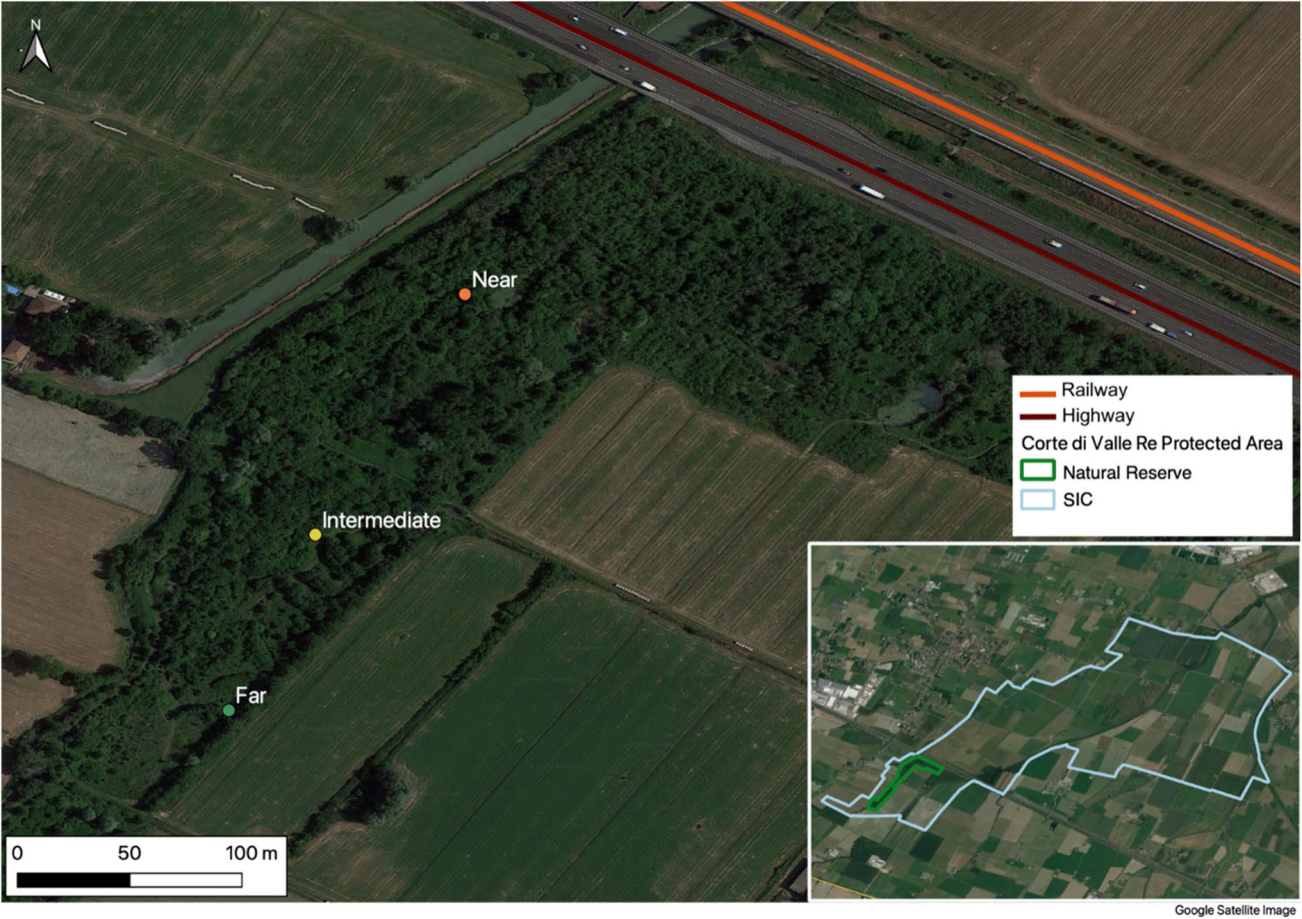
Map of the study site, the regional Natural Reserve Fontanili di Corte Valle Re, with the indication of the three sampling points in which the mist nets were located and the high-speed ways

Highways and railways can be considered the main sources of disturbance since the Reserve has few visitors per year and the trails are far from the nets. The seasonal presence of hunters in the surrounding areas may be considered an additional disturbance factor for the birds and may push them into the Reserve’s core. Distance from the Reserve and high-speed ways was 160 m from the nearest net (Near), 320 m from the intermediate one (Intermediate), and 425 m from the farthest one (Far) (Fig. 1). To ensure comparability, we checked that the three areas, although different in shape, have similar vegetation and are surrounded by the same type of agricultural field. One main difference among the three sampling sites is that the Near site is surrounded by a slightly higher forest cover, in an L shape, than the other two sites. We also confirmed that the three sampling points were located in areas with similar vegetation and environmental conditions.

Acoustic disturbance has been measured in the center of three transects using professional digital phonometers with a range of measures from 30 to 130 dBA and a sensitivity of 0.1 dBA, during optimal weather conditions, without wind or adverse meteorological conditions. The phonometers were set in the center of the three transects, detecting values at the same time for periods of 30 min and three repetitions during the day. We measured the acoustic disturbance in randomly selected days to provide empirical evidence of the differential acoustic impacts in the three sampling areas. We assumed that the acoustic disturbance throughout the year is similar to the average noise detected during the randomly selected days of recordings. We have good reasons to believe that our assumption is correct because the only variable factor is the wind and it is quite unpredictable during the seasons, while the railway and highway traffic noise remains almost constant throughout the year. The measurements returned 3573 records — two measurements every second — for every point, for a total of 10,719. To separate noise pollution caused respectively by railway and highway infrastructure, data were separated by calculating, for each study point, the value corresponding to the 95th percentile, dividing them into average noise values and peak noise values.

Bird captures were made using the standard procedure for birds’ ringing. The nets were opened before sunrise and monitored six times in the following hours. Ancillary data were collected including time of the catch, ring code, recapture, species name, Euring specific code, status, molt, plumage, weight, age, sex, fat, muscle, measurements of tarsus, chord, and third remiges’ length. The equipment included a bird identification guide, rings of various sizes, banding pliers, different rules, and a portable scale. Data has then been transferred to electronic datasets.

The statistical analysis, performed with the software R and ggplot package (R Core Team 2016), included the calculation of the AED index (*Absolute Effective Diversity*), to estimate the number of species observed in an area, including those not detected (Cazzolla Gatti et al. 2020). We also compared temporal trends, during years and seasons, between the three areas. To understand whether the acoustic disturbance affects species diversity and abundance Wilcoxon’s test (Wilcoxon 1992) was applied.

## Results

In the three sampling nets, 41 different species of birds were captured and ringed, and their relative abundance was recorded (Table 1). The three sampling sites showed differences in their richness and abundance, with higher values always for the Far net (Table 2). Some of the species protected under the Bird Directive 2009/147/CE have been ringed and collected in this study: the marsh warbler (*Acrocephalus palustris*), the pied flycatcher (*Ficedula hypoleuca)*, the red-backed shrike (*Lanius collurio*), the nightingale (*Luscinia megarhynchos*), and the song thrush (*Turdus philomelos*).

**Table 1:** List of bird species collected in the three sampling nets with their common name, scientific name, and abundance

Common name	Scientific name	Abundance			
		Near	Interm	Far	TOT
Blackbird	*Turdus merula*	46	38	28	112
Robin	*Erithacus rubecula*	58	85	103	246
Nightingale	*Luscinia megarhynchos*	31	72	45	148
Blackcap	*Sylvia atricapilla*	45	62	60	167
Redstart	*Phoenicurus phoenicurus*	1	0	16	17
Lesser whitethroat	*Sylvia curruca*	0	0	2	2
Pied flycatcher	*Ficedula hypoleuca*	0	0	5	5
Wryneck	*Jynx torquilla*	0	0	1	1
Goldfinch	*Carduelis carduelis*	0	2	2	4
Blue tit	*Cyanistes caeruleus*	5	18	11	34
Song thrush	*Turdus philomelos*	9	21	21	51
Tree pipit	*Anthus trivialis*	1	0	1	2
Long-tailed tit	*Aegithalos caudatus*	24	13	43	80
Great tit	*Parus major*	20	13	20	53
Firecrest	*Regulus ignicapillus*	0	1	4	5
Dunnock	*Prunella modularis*	3	4	2	9
Chiffchaff	*Phylloscopus collybita*	8	9	17	34
Jay	*Garrulus glandarius*	1	2	4	7
Wren	*Troglodytes troglodytes*	6	1	0	7
Hawfinch	*Coccothraustes coccothraustes*	4	0	0	4
Redwing	*Turdus iliacus*	0	0	1	1
Goldcrest	*Regulus regulus*	6	1	19	26
Greenfinch	*Chloris chloris*	0	1	0	1
Kestrel	*Falco tinnunculus*	0	1	0	1
Marsh warbler	*Acrocephalus palustris*	5	4	1	10
Great spotted woodpecker	*Dendrocopos major*	1	2	1	4
Red-backed shrike	*Lanius collurio*	0	2	0	2
Buzzard	*Buteo buteo*	0	0	1	1
Sparrowhawk	*Accipiter nisus*	0	0	2	2
Garden warbler	*Sylvia borin*	6	4	11	21
Spotted flycatcher	*Muscicapa striata*	0	0	1	1
Starling	*Sturnus vulgaris*	0	5	0	5
Willow warbler	*Phylloscopus trochilus*	0	2	1	3
Pheasant	*Phasianus colchicus*	0	1	0	1
Chaffinch	*Fringilla coelebs*	1	1	0	2
Scops owl	*Otus scops*	0	0	2	2
Long-eared owl	*Asio otus*	0	0	1	1
Short-toed treecreeper	*Certhia brachydactyla*	0	0	1	1
Serin	*Serinus serinus*	1	0	0	1
Melodious warbler	*Hippolais polyglotta*	0	0	1	1
Woodpigeon	*Columba palumbus*	0	0	1	1

**Table 2:** Diversity indices used for the calculation of the AED index: total abundance (N), observed species richness (S), Shannon index, and Simpson index

	Near	Intermediate	Far	Total
Total abundance (N)	282	365	429	1076
Species richness (S)	21	25	32	41
Shannon’s index	2.38	2.30	2.56	2.55
Simpson’s index	0.88	0.86	0.89	0.88
AED index	28.21	32.09	41.59	50.81
S.E. AED	7.16	6.89	8.56	8.04

**Fig. 2:**
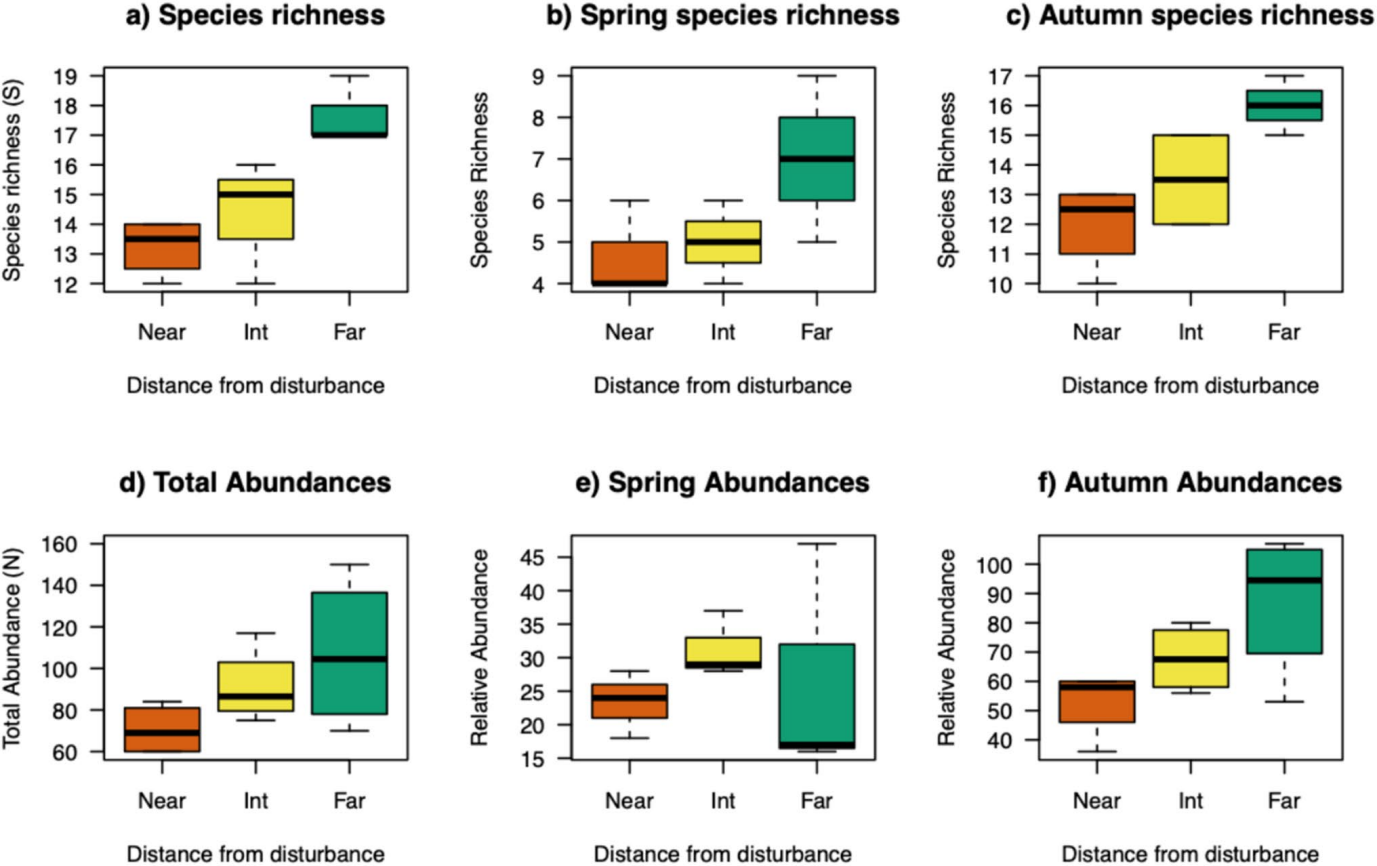
Bird species richness (S) and total abundance (N) in the whole study period (a and d, respectively), in Spring (b and e), and Autumn (c and f). The median (central line in the box) represents the values that fall between the second (lower quartile value) and the third quartile (upper quartile value). The whiskers include data that lie between the 10th percentile (lower bound) and the lower quartile value, and between the upper quartile and the 90th percentile. Points outside the lower and upper bounds are outliers

To better analyze the contribution of resident and migratory species to the total richness and abundance in the three sampling areas, we separate species collected in Spring from those collected in Autumn (Figure 2). In terms of species richness, the Far net shows higher values for both periods (*p* = 0.021 between Near and Far sampling points), whereas Spring abundance shows lower values in the Far net compared to the other sampling sites (*p* = 0.036).

**Fig. 3:**
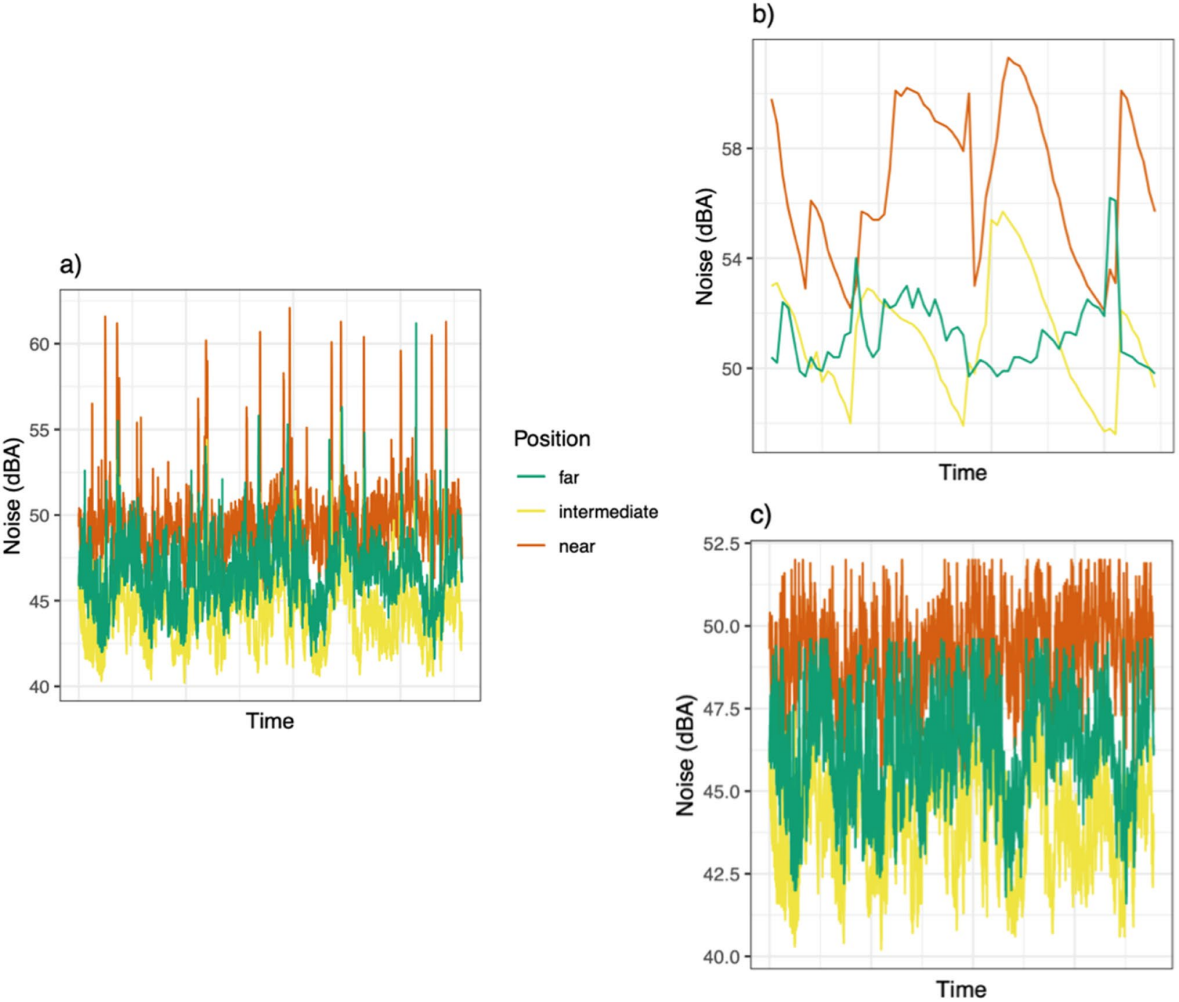
Time series of acoustic disturbances (in dB) (**a**) separated as peaks noise values (**b**) and average noise values (**c**). To separate the noise pollution caused respectively by railway and highway infrastructure, acoustic recordings were separated by calculating, for each sampling point, the value corresponding to the 95th percentile, dividing them into average noise values and peak noise values

**Fig. 4:**
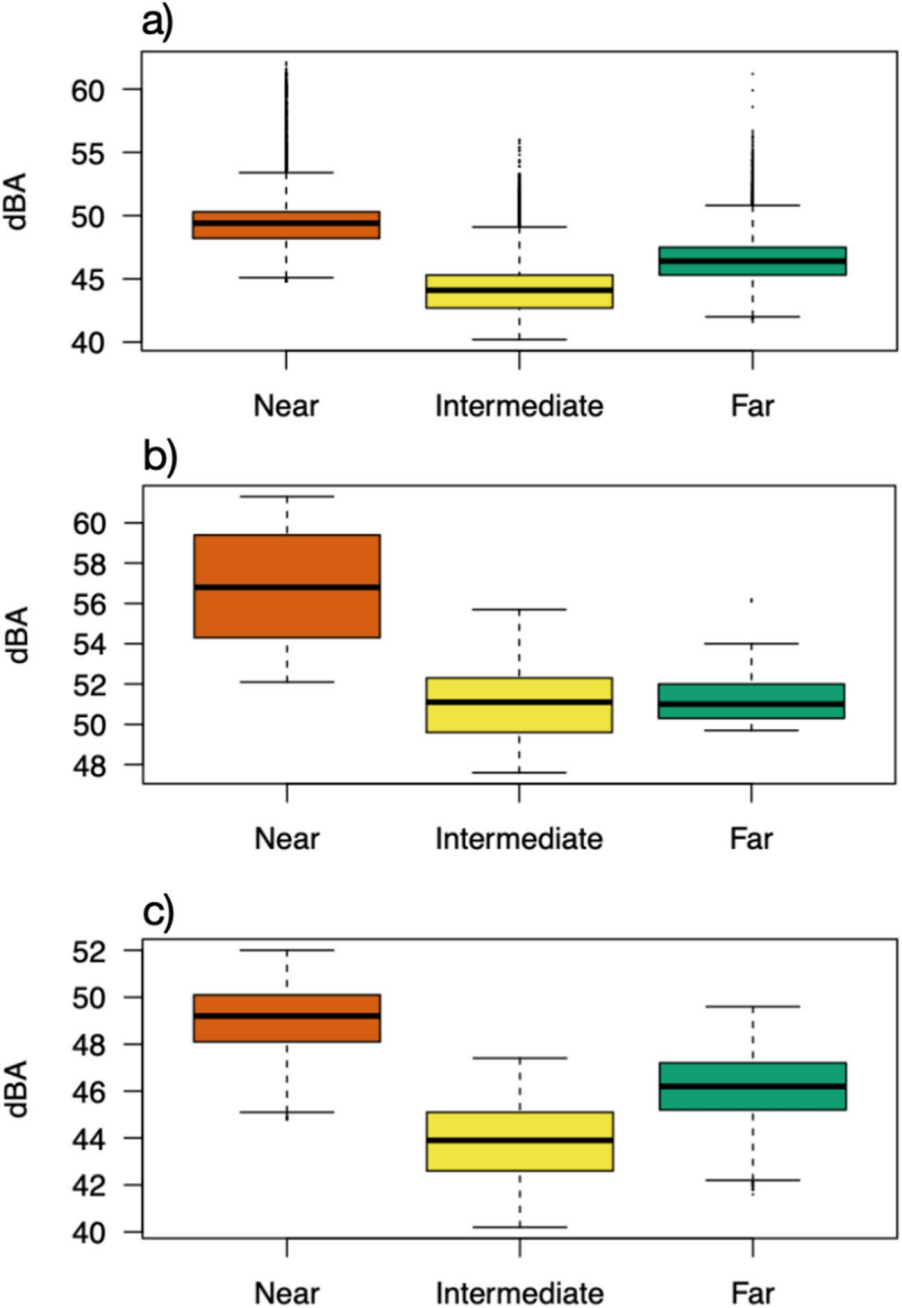
Noise pollution (in dB) distribution (**a**) separated into peak values distribution (**b**) and average values distribution (**c**). The median (central line in the box) represents the values that fall between the second (lower quartile value) and the third quartile (upper quartile value). The whiskers include data that lie between the 10th percentile (lower bound) and the lower quartile value, and between the upper quartile and 90th percentile. Points outside the lower and upper bounds are outliers

Time series of noise pollution recordings show a clear separation of the three sampling nets, with higher values (in dB) at the Near sampling point (Figure 3). Then, the time series were separated into peak noise values and average noise values. This separation of noise pollution allowed us to clearly show that the disturbance caused by the passage of high-speed trains (Fig. 3b) is scattered (because each recorded peak corresponded to a monitored time of train arrival), whereas the disturbance caused by the noise of motor vehicles on the highway is constant (Fig. 3c). In both cases, the Near sampling point recorded the highest noise pollution, which was higher than at the Intermediate and Far nets both for railways and highways (Fig. 4).

## Discussion

We analyzed the impact of a highway and a high-speed railway on the species diversity of a protected area in Italy, from autumn 2019 to autumn 2022.

Species richness decreases in the proximity of the sources of disturbance. In fact, bird diversity near the infrastructures is lower than the one observed in the more distant point, as confirmed also by previous studies: bird species richness is strongly affected by human disturbance such as agriculture, forest harvesting, roads, and urban and industrial areas in the surroundings of a habitat (Zhang et al. 2013). The significant differences between the nearest and farthest sampling points confirm our hypothesis of a higher impact due to high-speed ways, although the intermediate sampling net shows contrasting results attributable to the mid-domain effect that may lead to a higher abundance and diversity in the core of a reserve and a decrease of them at its edge (Colwell and Lees 2000).

In our study, bird abundance was significantly higher in the central area compared to the sampling point near the disturbance source. This is probably due to the mid-domain and edge effects, which lead the specimens to concentrate at the core of the Reserve, seeking protection in a less disturbed habitat. Bird diversity in the center of the Reserve was even a bit higher than at the farthest sampling point from noise pollution, and this may be because this point is close to the limit of the protected area and to the agricultural fields in which hunting seasonally takes place.

Total abundance shows a significant growing trend moving away from the source of disturbance, confirming the hypothesis that infrastructures represent the major reason for differences in bird species. Although a general increase is evident, it is not always linear and may reflect the different distribution of species within the reserve and their different behaviors. Birds that vocalize more could live further away from the source of noise pollution, which would interfere with the correct communication of individuals and consequently with their reproductive strategies, often based on an elaborate execution of the song, as already confirmed by different studies: with increasing background noise levels, males of European robin proved to be more likely to move away from the noise source and changed their singing behavior gradually (McLaughlin and Kunc 2013), and the number of birds per study area was shown to increase with the distance from the roads (Polak et al. 2013). Generally, highway noise has an impact on birds within several hundred meters (up to 1 km) from the source, despite visual disturbance and vehicular pollutants extended for a shorter distance from highways (Dooling and Popper 2016; Benítez-López A. et al. 2010).

In our sampling sites, we found a significantly higher bird abundance during autumn than spring and this may be due to migratory species that are probably less affected by anthropic noise while sedentary species are the most impacted. Anthropogenic noise pollution has been observed in other studies to play stronger effects on breeding birds than, in opposition to our results, wintering species, assuming these different responses may reflect species differences in acoustic communications (Wang et al. 2013; Catchpole et al. 2003). Breeding birds often need an elevated number of frequencies and types of acoustic contacts to complete the entire breeding cycle, such as mate attraction, territorial advertisement, or parent-offspring communication (Wang et al. 2013).

The absolute and effective diversity index (AED), which provides a comparable estimation of species missed from the sampling, confirms an important increase in the number of species moving away from high-speed ways. Some of these species could have different habitat preferences, while others, probably present in the reserve, have never been captured for random reasons. The index provided a value of potentially 50.81 living in the Reserve, which seems reliable as many species have been identified besides the 41 species detected in this study. The continuation of monitoring will certainly improve this work, allowing a higher bird species detection.

The highway A1 Milan–Naples and high-speed railway Milan–Bologna are highly busy causing a strong acoustic disturbance as measured by our phonometers. This may also be the reason why the reserve is rarely visited by people, as nature appreciation is enhanced by natural sounds and decreased by extraneous noises produced by road traffic, for instance (Chau et al. 2010).

Noise peaks in correspondence with the scattered passage of high-speed and regional trains seem to be less impacting for bird communication than the constant noise produced by the highway although both may importantly contribute to the acoustic disturbance.

It has been documented how the background acoustic disturbance — which grows exponentially in combination with the transit of high-speed trains or with the use of car horns — has repercussions on birdlife (Pickett et al. 1989; Parris and Schneider 2009; Lucas et al. 2017).

Reijnen et al. (1995) showed evidence that, in woodland, noise is probably the most critical factor in causing reduced densities close to roads. In regression analysis, using vehicle noise and visibility as response variables, the noise seemed to be the best and, in many species, the only predictor of observed depressed densities in the proximity of the road. Similarly, in open agricultural fields, several species of breeding birds showed a density reduction of almost 100% due to dense traffic, possibly causing an important loss of species richness (Reijnen et al. 1997), with total population density reduced by 39% in open agricultural grasslands and 35% in woodlands. Several studies also on non-bird species support the finding that, generally, animals change their distributions in response to anthropogenic noise, as the observation of Sonoran pronghorn’s behavior in avoiding loud areas due to military jet overflights (Barber et al. 2009). High levels of noise have been shown to affect wildlife physiological aspects, including temporary and permanent hearing loss (Barber et al. 2009).

In light of our findings and previous studies, to better protect birdlife and wildlife in general, it would be important to limit the extent of the disturbance by setting anti-noise barriers alongside the infrastructures. Most of the scientific literature is in line in acknowledging that anti-noise walls can reduce acoustic pollution of 5-15 dB: the volume of the sound pressure which a wall can absorb is directly proportional to the size of the sound pressure of the incoming source, considering that the decibel scale is logarithmic, even a small numeric variations translate into a major changes in sound perception (Hranický et al. 2016). According to Adamec et al. (2011), on average, well-designed barriers reduce noise by approximately 4 or more dB, depending on the geometry of traffic noise propagation.

Different types of noise barriers can be considered a valid help: reflecting walls with a smooth surface or absorbing walls with rugged folds are useful not only for the reflection of sound waves but also for their direct limitation; however, it is important to note that reflective barriers are often made with transparent materials and may be a danger for birds if not appropriate visual deterrent is placed (Hranický et al. 2016). Another related issue can occur where noise barriers are built only on one side of the track and animals can remain trapped on the infrastructure, but a simple solution can be the placement of stickers with dark contours of predatory birds for deterrence and further the integration of these structures with elevated ecological corridors (Hranický et al. 2016). In this specific case, noise barriers should reach a maximum height equivalent to the height of the tree crowns, as they could otherwise create an additional obstacle to the passage of moving birds.

Overall, our study was conducted in a relatively longer timespan (4 years) than most of the previous studies (Reijen et al. 2006; Wiacek et al. 2015) and, therefore, provides reliable evidence that road and rail traffic, particularly on high-speed ways, is one of the main causes of the loss of bird diversity, even in protected areas.

## Data Availability

The data that support the findings of this study are available from the corresponding author, SP, upon reasonable request.
